# Inverse correlation between Ki67 expression as a continuous variable and outcomes in luminal HER2-negative breast cancer

**DOI:** 10.20407/fmj.2018-021

**Published:** 2019-04-17

**Authors:** Kaori Ushimado, Naomi Kobayashi, Masahiro Hikichi, Tetsuya Tsukamoto, Makoto Urano, Toshiaki Utsumi

**Affiliations:** 1 Department of Breast Surgery, Fujita Health University, School of Medicine, Toyoake, Aichi, Japan; 2 Department of Diagnostic Pathology, Fujita Health University, School of Medicine, Toyoake, Aichi, Japan

**Keywords:** Breast cancer, Ki67, Distant disease-free survival, Overall survival

## Abstract

**Objectives:**

Few studies to date have investigated the prognostic significance of Ki67 expression as a continuous variable in breast cancer. This study aimed to evaluate the impact of Ki67 expression as a dichotomous or continuous variable on outcomes in estrogen receptor (ER)+ and human epidermal growth factor receptor 2 (HER2)– breast cancer.

**Methods:**

Survival analysis was performed to estimate the likelihood of distant recurrence and death in retrospective data from 794 patients with ER+/HER2– breast cancer. We assessed the relationship between outcomes and two Ki67 cutoffs, 14% and 20%, and the Ki67 labeling index as a continuous variable.

**Results:**

In univariate analysis, T stage, lymph node involvement, histological grade, progesterone receptor status, and Ki67 expression at the two cutoffs and as a continuous variable were identified as significant prognostic factors for distant disease-free survival (DDFS) and overall survival (OS). There were no statistical differences in DDFS and OS between women with Ki67 expression of <14% and 14–<20%. Multivariate analysis showed that Ki67 expression ≥20% was an independent prognostic indicator for DDFS. Regarding the risk of distant metastasis, the 20% cutoff was more reliable than 14%. We also found that Ki67 expression as a continuous variable was an independent prognostic factor for DDFS and OS in multivariate analyses.

**Conclusions:**

High Ki67 expression is associated with a survival disadvantage in patients with ER+/HER2– breast cancer, indicating that these patients might have a higher risk of recurrence after primary treatment and might therefore benefit from individualized treatment.

## Introduction

Breast cancer is the most common malignancy for women in many countries.^1^ Recent microarray studies of gene expression have demonstrated that breast cancer is a molecularly heterogeneous assemblage of different subtypes characterized by distinct aberrations at the molecular level.^2,3^ Breast cancer can be classified into at least five distinct subtypes: luminal A, luminal B, human epidermal growth factor receptor 2 (HER2) overexpressing, basal-like, and normal breast. Multiple studies have shown that protein expression can act as a surrogate for the genomic profile of breast cancer when classifying breast cancer into subtypes with distinct biological characteristics and clinical outcomes.^4,5^ Estrogen receptor (ER), progesterone receptor (PgR), and HER2 are the best-characterized biomarkers associated with targeted therapy.^6^ Ki67 is a nuclear protein that correlates with cellular proliferation,^7^ and has been widely explored as a proliferation marker to determine the degree of growth and prognosis of various cancers.^8–21^ Recently, the prognostic and predictive importance of Ki67 expression in breast cancer has been highlighted. The St. Gallen international expert consensus statement includes treatment algorithms based on the classification of breast cancer subtypes according to immunohistochemistry results for Ki67 expression as well as for ER, PgR, and HER2.^22,23^ The European Society for Medical Oncology (ESMO) clinical practice guidelines also describe the usefulness of Ki67 in daily clinical practice for ER+/HER2– breast cancer.^24^ However, the American National Comprehensive Cancer Network guidelines for breast cancer do not include the assessment of Ki67 or its role in therapeutic decision-making.^25^ Thus, the usefulness of Ki67 in decision-making on treatment for ER+/HER2– breast cancer remains under discussion.

The cutoff point between high and low Ki67 labeling index for dividing patients with ER+/HER2– breast cancer into two distinct biological or prognostic different groups is still a matter of debate. Moreover, few studies have examined the impact of the Ki67 labeling index as a continuous variable for prognosis in operable breast cancer.^21^ Thus, the relationship between Ki67 expression as a dichotomous or continuous variable and outcomes is not yet fully understood. In the present study, we examined the relationship among Ki67 expression as a dichotomous or continuous variable, clinicopathological characteristics, and outcomes in patients with ER+/HER2– breast cancer. The aim of this study, which focused on distant disease-free survival (DDFS) and overall survival (OS), was to evaluate differences in Ki67 expression and outcomes among patients with ER+/HER2– breast cancer.

## Methods

### Patients

We retrospectively examined data from 794 women with ER+/HER2– breast cancer treated at Fujita Health University Hospital between January 2003 and December 2014. Patients with stage IV or bilateral disease and occult or noninvasive cancer were excluded from this study. Male patients with breast cancer and patients lost to follow-up immediately after surgery were also excluded. Histological grade was determined based on the Bloom and Richardson classification system.^26^ Indications for chemotherapy generally included tumors that were hormone receptor negative, HER2 positive, triple negative (ER negative, PgR negative, and HER2 negative), or node positive, or had higher histological grade or high Ki67 expression. We collected clinicopathological data from the medical records of eligible patients.

With regards to Ki67, we used two cutoffs, 14%^15,16,21^ and 20%,^12,14,18,19,21^ where the Ki67 labeling index was considered a dichotomous variable as well as a continuous variable for survival analyses. We investigated the relationship between Ki67 expression (14% and 20% cutoffs) and clinicopathological factors (age, stage, T stage, pathological node status, histological grade, PgR status, chemotherapy, endocrine therapy, and type of surgery). The primary outcomes of the study were first distant recurrence and death from any cause. DDFS and OS were calculated from the date of diagnosis to the date of distant recurrence or death and to the date of death from any cause, respectively.^27^ We investigated the prognostic factors for DDFS and OS in univariate and multivariate analyses, and selected multiple covariates [Ki67 (Ki67 cutoff and the Ki67 labeling index as a continuous variable), T stage, pathological node status, histological grade, and PgR status]. This retrospective study was approved by the Ethics Committee of Fujita Health University (reference no. HM16-138).

### Immunohistochemistry

Immunohistochemical methods were as previously described.^28^ Immunohistochemical staining was performed for ER and PgR using the SP1 and the 1E2 (Ventana Medical, Tucson, AZ, USA) staining systems, respectively. Positive ER or PR status was defined as the presence of ≥1% positive cancer cells. Immunohistochemical assays for HER2 status was determined using the Pathway anti-HER2/neu test (Ventana Medical). Fluorescence in situ hybridization (FISH) was performed using the PathVysion HER-2 DNA probe kit (Abbott France SAS, Rungis, France). An immunohistochemical result of 3+ or FISH amplification was defined as a positive result. Ki67 staining was performed using a MIB-1 monoclonal antibody (Dako, Glostrup, Denmark). At least 1000 invasive cells were scored and the Ki67 labeling index was expressed as the percentage of positively stained cells among the total number of invasive cells. Although surgical specimens were used as sample sources, core biopsies before neoadjuvant therapy were used for patients who underwent neoadjuvant therapy. All markers were assessed with blinding to clinical data.

### Statistical analysis

Statistical analyses were performed using SPSS 22.0 software (IBM Corp., Armonk, NY, USA). The chi-square test was used for contingency table analysis. Survival curves were generated using the Kaplan–Meier method.^29^ Comparisons of survival between groups were performed using the log-rank test. Cox regression analyses were performed for DDFS and OS to calculate crude and adjusted hazard ratios (HRs) and 95% confidence intervals (CIs) for various groups.

## Results

### Clinical characteristics of ER+/HER2– breast cancers by Ki67 cutoff

Table 1 shows the clinical profile of the 794 patients with ER+/HER2– breast cancer stratified by the Ki67 labeling index at cutoffs of 14% and 20%. Patients with high Ki67 expression were significantly younger than those with low Ki67 expression. At a cutoff of 14%, the proportion of patients aged <40 years was 4.2% in the low Ki67 expression group versus 14.6% in the high Ki67 expression group (p<0.001). With a cutoff of 20%, the proportion of patients aged <40 years was 6.7% in the low Ki67 expression group versus 14.8% in the high Ki67 expression group (p<0.001). Low Ki67 expression was significantly associated with earlier disease stage (14% cutoff: 62.7% of patients in the low Ki67 expression group had Stage I disease vs. 34.4% in the high Ki67 expression group, p<0.001; 20% cutoff: 55.2% vs. 35.5%, p<0.001) and earlier T stage (14% cutoff: 64.4% in the low Ki67 expression group had T1 stage vs. 37.8% in the high Ki67 expression group, p<0.001; 20% cutoff: 57.6% vs. 38.3%, p<0.001).

Among the 794 patients, data on pathological node status was missing for 30 patients. Of these 30 patients, 26 did not undergo axillary surgery. The remaining four patients had no pathological node involvement after neoadjuvant chemotherapy and had no evidence of negative lymph node status before neoadjuvant chemotherapy. A higher proportion of patients with low Ki67 expression had node-negative disease (14% cutoff: 67.9% in the low Ki67 expression group vs. 54.5% in the high Ki67 expression group, p<0.001; 20% cutoff: 65.1% vs. 53.5%, p<0.001). Data on histological grade were not available for 17 patients. Lesions with low Ki67 expression were more likely to be of lower histological grade (14% cutoff: grade 1, 49.4% vs. 13.1%; grade 2, 46.4% vs. 55.8%; grade 3, 1.5% vs. 29.6%, p<0.001; 20% cutoff: grade 1, 40.0% vs. 14.1%; grade 2, 44.8% vs. 64.1%; grade 3, 12.6% vs. 20.7%, p<0.001). Patients with high Ki67 expression received chemotherapy more frequently than patients with low Ki67 expression (14% cutoff: 19.1% in the low Ki67 expression group vs. 52.3% in the high Ki67 expression group, p<0.001; 20% cutoff: 27.6% vs. 51.8%, p<0.001).

We also investigated the relationship between surgical treatment and Ki67 expression. The rate of breast-conserving surgery (BCS) in patients with low Ki67 expression was significantly higher than in those with high Ki67 expression (14% cutoff: 68.1% of patients in the low Ki67 expression group underwent BCS vs. 58.9% in the high Ki67 expression group, p=0.007; 20% cutoff: 67.1% vs. 56.3%. p=0.003). In addition, patients with high Ki67 expression underwent axillary lymph node dissection (ALND) more frequently than patients with low Ki67 expression (14% cutoff: 31.1% of patients in the low Ki67 expression group underwent ALND vs. 39.8% in the high Ki67 expression group, p=0.005; 20% cutoff: 32.5% vs. 41.4%. p=0.015).

### DDFS and OS by Ki67 expression level

**Figure 1:**
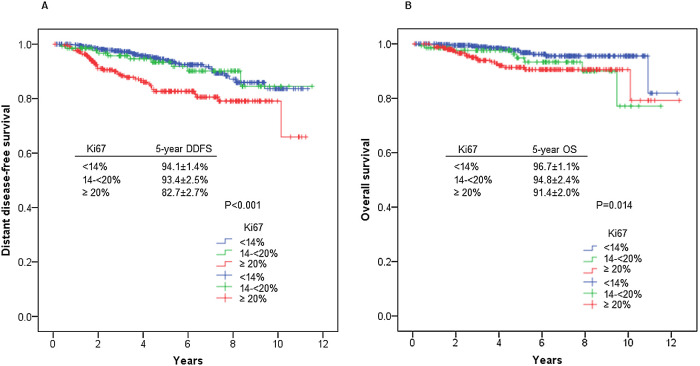
Distant disease-free (A) and overall survival (B) in 794 women with breast cancer according to Ki67 expression level.

The estimated 5-year DDFS rate was 94.1±1.4% for women with Ki67 expression <14%, 93.4±2.5% for women with Ki67 expression of 14–<20%, and 82.7±2.7% for women with Ki67 expression ≥20% (p<0.001) (Figure 1A). The estimated 5-year OS rate was 96.7±1.1% for women with Ki67 expression <14%, 94.8±2.4% for women with Ki67 expression of 14–<20%, and 91.4±2.0% for women with Ki67 expression ≥20% (p=0.014) (Figure 1B).

### Univariate and multivariate survival analysis

In the univariate analysis, T stage, lymph node involvement, histological grade, PgR status, and Ki67 expression (Ki67 cutoff and the Ki67 labeling index as a continuous variable) were significant prognostic factors for DDFS and OS (Table 2). In the univariate analysis, there were no statistical differences in DDFS and OS between women with Ki67 expression <14% versus 14–<20% (Table 2). Table 3 shows the results of multivariate analysis of DDFS and OS by Ki67 status. At the Ki67 cutoff of 14%, T2–4 stage and nodal involvement remained associated with DDFS and OS, but Ki67 was not associated with DDFS or OS (Table 3A). At the Ki67 cutoff of 20%, T2–4 stage, nodal involvement, negative PgR status, and Ki67 expression ≥20% remained associated with DDFS, but T2–4 stage and Ki67 expression ≥20% were not associated with OS (Table 3B). Furthermore, T stage, pathological node status, PgR status, and Ki67 expression as a continuous variable were prognostic factors for DDFS, but among these, only T stage was not associated with OS (Table 3C).

## Discussion

Gene expression profiling studies have shown that HER2-negative hormone receptor-positive breast cancer can be classified into two biologically distinct subtypes: luminal A and luminal B.^2,3^ Moreover, the importance of Ki67 in breast cancer has become increasingly apparent after Cheang et al. showed that the Ki67 labeling index is useful in distinguishing between the luminal A and luminal B subtypes.^4^ The clinical application of a Ki67 cutoff in breast cancer has been extensively investigated,^11–21^ but few studies to date have examined the clinical significance of Ki67 expression as a continuous variable.^21^ In the present study, we explored the clinical characteristics and outcomes of a retrospective cohort of patients using Ki67 expression as a dichotomous or continuous variable. Ki67 cutoffs of 14% and 20% and the Ki67 labeling index as a continuous variable were associated with poor prognosis in women with ER+/HER2– breast cancer in the univariate analysis.

The optimal cutoff of Ki67 to distinguish between the luminal A and luminal B subtypes in clinical use remains a matter of broad discussion. In previous studies, cutoffs of 10%,^11,13,20^ 14%,^15,16,21^ and 20%^12,14,18,19,21^ have been evaluated. The 2011 St. Gallen consensus statement proposed using a 14% cutoff for distinguishing between the luminal A and B subtypes^22^ based on the study by Cheang et al.^4^ Two years later, revisions to the recommendations included laboratory-specific cutoffs and a cutoff of 20%.^23^ ESMO guidelines also refer to a cutoff of 20%.^24^ Our results showed that patients with low Ki67 expression generally had more favorable outcomes than those with high Ki67 expression (Figure 1A, Figure 1B), but there were no statistical differences in DDFS and OS between women with Ki67 expression <14% versus 14–<20% (Table 2). When considered together in the multivariate analysis, nodal status was a stronger predictor of OS. The 14% cutoff did not provide additional significant prognostic information on survival. Multivariate analysis further showed that a Ki67 cutoff of 20% was an independent prognostic indicator for DDFS. As a consequence, when assessing the risk of distant metastasis, 20% may be a more reliable cutoff than 14%. We also found that the Ki67 labeling index as a continuous variable was an independent prognostic factor for DDFS and OS in the multivariate analysis. Our findings are consistent with the results of Gallardo et al.^21^ Thus, the Ki67 labeling index might play a prognostic role in luminal HER2-negative breast cancer.

Given that Ki67, T stage, pathological node status, histological grade, and PgR are established as important prognostic factors for breast cancer, they were included as covariates in the univariate and multivariate analyses. We excluded body mass index and comorbidity as covariates as they were not precisely recorded in the medical records. Risk reduction for DDFS and OS can vary according to the type of chemotherapy administered.^30,31^ As multiple types of chemotherapy were administrated to the included patients, we also excluded chemotherapy as a covariate.

We found that cancers with low Ki67 expression were smaller, more frequently node-negative, and more frequently of lower histological grade and earlier stage than cancers with high Ki67 expression. These findings are consistent with results from previous studies.^14,17,20^ High Ki67 expression indicates high proliferative activity; thus, tumors with a higher percentage of cells expressing Ki67 might grow faster and be more aggressive. We speculate that since slow-growing tumors generally have a longer asymptomatic period than faster-growing tumors, tumors with high Ki67 expression are more likely to be detected at a more advanced stage.

Our study had certain limitations. First, the study was retrospective in design, with data collected at a single institution; thus, it had potential biases inherent to all retrospective studies, such as selection bias. Second, the number of patients was moderate, meaning that the results must be interpreted with caution for clinical use because a moderate sample size might not yield conclusive results. A larger observational series could provide additional data.

However, our study also contains strengths. Few studies to date have evaluated the prognostic importance of Ki67 expression as a continuous variable; most have considered the prognostic importance of Ki67 expression as a dichotomous variable. We found a good correlation between Ki67 expression as a continuous variable and DDFS and OS. Multivariate analysis further showed that Ki67 expression was an independent prognostic indicator for DDFS and OS. Higher expression of Ki67 in breast cancer is associated with worse prognosis. Prediction of prognosis has historically been guided by disease extension, as indicated by tumor stage, for example, but it has become clearer that tumor biology is more relevant to prognosis than tumor size.^32^ Although cancers with high Ki67 expression are typically at a more advanced stage than cancers with low Ki67 expression, multivariate analysis showed that Ki67 expression as a continuous variable was an independent prognostic factor for DDFS and OS. In prognostic studies using cutoffs with dichotomous variables, the prognostic impact could vary depending on the cutoff point. Our findings indicate that Ki67 plays an important role in tumor biology and can influence prognosis.

In conclusion, patients with luminal HER2-negative breast cancer and high Ki67 expression have a survival disadvantage, and may have a higher risk of recurrence after primary treatment. Thus, prospective therapeutic approaches and management, such as patient-tailored treatment strategies, should be considered for this patient population.

## Figures and Tables

**Table1:** Clinical profile of patients with ER+/HER2– breast cancer according to two Ki67 cutoffs (n=794)

	Ki67 <14%	Ki67 ≥14%	*p* value	Ki67 <20%	Ki67 ≥20%	*p* value
Number of patients	405	389		538	256	
Age (years)						
<40	17 (4.2%)	57 (14.6%)		36 (6.7%)	38 (14.8%)	
40–49	98 (24.2%)	95 (24.4%)		116 (21.6%)	77 (30.1%)	
50–59	98 (24.2%)	78 (20.1%)		127 (23.6%)	49 (19.1%)	
60–69	107 (26.4%)	93 (23.9%)		149 (27.7%)	51 (19.9%)	
70–79	85 (21.0%)	66 (17.0%)	<0.001	110 (20.4%)	41 (16.0%)	<0.001
Stage						
I	254 (62.7%)	134 (34.4%)		297 (55.2%)	91 (35.5%)	
IIA	107 (26.4%)	153 (39.3%)		165 (30.7%)	95 (37.1%)	
IIB	21 (5.2%)	66 (17.0%)		45 (8.4%)	42 (16.4%)	
IIIA	6 (1.5%)	14 (3.6%)		8 (1.5%)	12 (4.7%)	
IIIB	17 (4.2%)	18 (4.6%)		22 (4.1%)	13 (5.1%)	
IIIC	0 (0%)	4 (1.0%)	<0.001	1 (0.2%)	3 (1.2%)	<0.001
T stage						
T1	261 (64.4%)	147 (37.8%)		310 (57.6%)	98 (38.3%)	
T2–4	144 (35.6%)	242 (62.2%)	<0.001	228 (42.4%)	158 (61.7%)	<0.001
Pathological node status						
Negative	275 (67.9%)	212 (54.5%)		350 (65.1%)	137 (53.5%)	
Positive	110 (27.2%)	167 (42.9%)		162 (30.1%)	115 (44.9%)	
Unknown	20 (4.9%)	10 (2.6%)	<0.001	26 (4.8%)	4 (1.6%)	<0.001
Histological grade						
1	200 (49.4%)	51 (13.1%)		215 (40.0%)	36 (14.1%)	
2	188 (46.4%)	217 (55.8%)		241 (44.8%)	164 (64.1%)	
3	6 (1.5%)	115 (29.6%)		68 (12.6%)	53 (20.7%)	
Unknown	11 (2.7%)	6 (1.5%)	<0.001	14 (2.6%)	3 (1.2%)	<0.001
PgR						
Negative	62 (15.3%)	53 (13.6%)		74 (13.8%)	41 (16.0%)	
Positive	343 (84.7%)	336 (86.4%)	0.500	464 (86.2%)	215 (84.0%)	0.397
Chemotherapy						
Given	77 (19.1%)	203 (52.3%)		148 (27.6%)	132 (51.8%)	
Not given	327 (80.9%)	185 (47.7%)	<0.001	389 (72.4%)	123 (48.2%)	<0.001
Endocrine therapy						
Given	390 (96.3%)	315 (81.4%)		464 (86.2%)	241 (94.9%)	
Not given	15 (3.7%)	72 (18.6%)	<0.001	74 (13.8%)	13 (5.1%)	<0.001
Breast surgery						
BCS	276 (68.1%)	229 (58.9%)		361 (67.1%)	144 (56.3%)	
Mastectomy	129 (31.9%)	160 (41.1%)	0.007	177 (32.9%)	112 (43.8%)	0.003
Axillary surgery						
No axillary surgery	19 (4.7%)	7 (1.8%)		22 (4.1%)	4 (1.6%)	
ALND±SNB	126 (31.1%)	155 (39.8%)		175 (32.5%)	106 (41.4%)	
SNB	260 (64.2%)	227 (58.4%)	0.005	341 (63.4%)	146 (57.0%)	0.015

Abbreviations: ALND, axillary lymph node dissection; BCS, breast-conserving surgery; PgR, progesterone receptor; SNB, sentinel lymph node biopsy.

**Table2:** Univariate analysis of distant disease-free survival and overall survival

Covariate	Distant disease-free survival				Overall survival		
	Hazard ratio	(95% CI)	*p* value		Hazard ratio	(95% CI)	*p* value
Ki67							
<14%	1.00				1.00		
≥14%	1.93	(1.22–3.06)	0.005		2.60	(1.32–5.14)	0.006
Ki67							
<20%	1.00				1.00		
≥20%	2.31	(1.48–3.60)	<0.001		2.18	(1.16–4.09)	0.015
Ki67							
<14%	1.00				1.00		
14–<20%	1.12	(0.54–2.30)	0.762		2.23	(0.91–5.49)	0.080
≥20%	1.54	(1.21–1.96)	<0.001		1.67	(1.16–2.39)	0.006
Ki67 as a continuous variable (per percentage gain)							
Ki67 percentage	1.03	(1.01–1.04)	<0.001		1.04	(1.02–1.05)	<0.001
T stage							
T1	1.00				1.00		
T2–4	3.22	(1.92–5.40)	<0.001		3.28	(1.55–6.90)	0.002
Pathological node status							
Negative	1.00				1.00		
Positive	3.52	(2.19–5.68)	<0.001		4.04	(2.04–8.02)	<0.001
Histological grade							
1	1.00				1.00		
2, 3	2.21	(1.23–3.95)	0.008		2.50	(1.04–6.00)	0.041
PgR							
Positive	1.00				1.00		
Negative	2.13	(1.29–3.51)	0.003		2.77	(1.42–5.40)	0.003

Abbreviations: BCS, breast-conserving surgery; CI, confidence interval; PgR, progesterone receptor.

**Table3:** Multivariate Cox analysis of distant disease-free survival and overall survival by Ki67 expression level at two cutoff values or as a continuous variable

A) 14% cutoff							
Covariate	Distant disease-free survival				Overall survival		
	Hazard ratio	(95% CI)	*p* value		Hazard ratio	(95% CI)	*p* value
T stage							
T1	1.00				1.00		
T2–4	2.29	(1.29–4.05)	0.005		2.01	(0.87–4.63)	0.100
Pathological node status							
Negative	1.00				1.00		
Positive	2.59	(1.56–4.29)	<0.001		2.84	(1.35–6.00)	0.006
Histological grade							
1	1.00				1.00		
2, 3	1.34	(0.71–2.53)	0.362		1.23	(0.49–3.11)	0.662
PgR							
Positive	1.00				1.00		
Negative	1.77	(1.06–3.14)	0.29		2.35	(1.14–4.87)	0.021
Ki67 expression							
<14%	1.00				1.00		
≥14%	1.54	(0.91–2.60)	0.109		2.11	(0.94–4.73)	0.069

B) 20% cutoff							
Covariate	Distant disease-free survival				Overall survival		
	Hazard ratio	(95% CI)	*p* value		Hazard ratio	(95% CI)	*p* value
T stage							
T1	1.00				1.00		
T2–4	2.23	(1.26–3.96)	0.006		2.14	(0.93–4.95)	0.74
Pathological node status							
Negative	1.00				1.00		
Positive	2.54	(1.53–4.23)	<0.001		2.87	(1.36–6.09)	0.006
Histological grade							
1	1.00				1.00		
2, 3	1.38	(0.74–2.57)	0.309		1.42	(0.57–3.53)	0.445
PgR							
Negative	1.00				1.00		
Positive	1.77	(1.03–3.05)	<0.001		2.32	(1.12–4.80)	0.024
Ki67 expression							
<20%	1.00				1.00		
≥20%	1.82	(1.12–2.95)	0.015		1.54	(0.77–3.07)	0.224

C) Continuous variable							
Covariate	Distant disease-free survival				Overall survival		
	Hazard ratio	(95% CI)	*p* value		Hazard ratio	(95% CI)	*p* value
T stage							
T1	1.00				1.00		
T2–4	2.25	(1.27–3.98)	0.005		1.97	(0.86–4.51)	0.11
Pathological node status							
Negative	1.00				1.00		
Positive	2.60	(1.57–4.32)	<0.001		2.87	(1.36–6.04)	0.006
Histological grade							
1	1.00				1.00		
2, 3	1.33	(0.71–2.49)	0.378		1.19	(0.47–3.00)	0.710
PgR							
Negative	1.00				1.00		
Positive	1.83	(1.06–3.14)	0.029		2.38	(1.15–4.92)	0.019
Ki67 as a continuous variable	1.00				1.00		
Each percentage gain	1.02	(1.003–1.032)	0.016		1.03	(1.01–1.05)	0.002

Abbreviation: CI, confidence interval; PgR, progesterone receptor.
